# miR-146b-5p functions as a tumor suppressor by targeting TRAF6 and predicts the prognosis of human gliomas

**DOI:** 10.18632/oncotarget.4895

**Published:** 2015-08-06

**Authors:** Jing Liu, Jinling Xu, Huining Li, Cuiyun Sun, Lin Yu, Yanyan Li, Cuijuan Shi, Xuexia Zhou, Xiuwu Bian, Yifang Ping, Yanjun Wen, Shujun Zhao, Hui Xu, Linlin Ren, Tongling An, Qian Wang, Shizhu Yu

**Affiliations:** ^1^ Department of Neuropathology, Tianjin Neurological Institute, Tianjin Medical University General Hospital, Tianjin 300052, China; ^2^ Tianjin Key Laboratory of Injuries, Variations and Regeneration of the Nervous System, Tianjin 300052, China; ^3^ Key Laboratory of Post-trauma Neuro-repair and Regeneration in Central Nervous System, Ministry of Education, Tianjin 300052, China; ^4^ Department of Biochemistry, Basic Medical College of Tianjin Medical University, Tianjin 300070, China; ^5^ Institute of Pathology and Southwest Cancer Center, Southwest Hospital, Third Military Medical University, Chongqing 400038, China; ^6^ Laboratory of Hormone and Development, Ministry of Health, Institute of Endocrinology, Tianjin Medical University, Tianjin 300070, China

**Keywords:** gliomas, miR-146b-5p, TRAF6, proliferation, apoptosis, prognosis

## Abstract

Down-regulation of miR-146b-5p contributes to tumorigenesis in several human cancers. However, the relevance of miR-146b-5p to prognosis, proliferation and apoptosis in gliomas remains unknown. In the present study, we demonstrated that miR-146b-5p expression was inversely correlated with grades and Ki-67 index in 147 human glioma specimens, but positively correlated with patients’ survival. Furthermore, two distinct subgroups of patients with grade I-IV gliomas with different prognoses were identified according to miR-146b-5p expression in our specimens. Cox regression showed that miR-146b-5p was an independent predictor for patients’ survival. Overexpression of miR-146b-5p dramatically suppressed glioma cell proliferation and induced apoptosis. Mechanistically, we validated TRAF6 as a direct functional target of miR-146b-5p and found that miR-146b-5p overexpression significantly decreased phosphorylated TAK1 and IκBα, the pivotal downstream effectors of TRAF6. Moreover, TRAF6 expression was positively correlated with glioma grades and Ki-67 index but inversely correlated with miR-146b-5p expression and predicted poor prognosis of glioma patients. In glioblastoma cell lines, silencing of TRAF6 could mimic the anti-tumor effect of miR-146b-5p. Our findings identify miR-146b-5p as a tumor suppressor and novel prognostic biomarker of gliomas, and suggest miR-146b-5p and TRAF6 as potential therapeutic candidates for malignant gliomas.

## INTRODUCTION

Gliomas are the most common primary brain tumors. The grade III and IV (malignant) gliomas, such as glioblastoma, are aggressive and lethal malignant neoplasms [1, 2]. Despite efforts being made to improve therapeutic strategies, the average survival of malignant glioma patients has been improved only slightly in the past decades [3]. Although malignant glioma patients tend to have poor prognosis, significant intragroup variations in their survival have been observed [4, 5]. This finding prompts that current histopathological criteria for glioma diagnosis are not suitable for comprehensive assessment of the patients’ status, especially their survival [6, 7]. Therefore, the new approaches of diagnosis, prognostic evaluation and therapy used for gliomas must be explored by understanding their genetic and epigenetic changes [8–11]. Recent studies have identified the molecular subtypes and a few prognostic biomarkers of glioblastoma [12–14]. However, the pathogenesis and prognostic signatures of gliomas have not been fully characterized.

miRNAs have recently been recognized as important regulators of cancer biologic behavior and the prognostic biomarkers [15–17]. Previous studies have shown that miR-146b-5p is associated with the occurrence and progression of some tumors, but its roles may be totally opposite in different tumors [18–23]. In papillary thyroid carcinoma, it acts as an oncogene and has been regarded as a relevant diagnostic marker [18–20], whilst in breast cancer, prostate cancer and gliomas, it shows the potency of a tumor suppressor [21–23]. Recent study has confirmed that miR-146b-5p downexpression predicts poor outcome in diffuse large B-cell lymphoma [24]. However, the prognostic significance of miR-146b-5p in gliomas remains unknown due to the lack of large pools of clinical specimens for screening.

The inhibition of apoptosis, uncontrollable proliferation, and invasive behavior of tumor cells are responsible for the deadly nature of malignant gliomas [3]. TRAF6, an E3 ubiquitin ligase, has recently been discovered to promote oncogenesis through inhibiting apoptosis and stimulating proliferation and invasion [25, 26]. Our previous study has discovered that miR-146b-5p expression is decreased in gliomas and that miR-146b-5p overexpression may suppress the migration and invasion of glioma cells by directly targeting MMP16 [27]. To our knowledge, however, it is not reported whether miR-146b-5p suppresses glioma cell proliferation and induces apoptosis through directly targeting TRAF6.

In the present study, we report for the first time that miR-146b-5p is a novel prognostic biomarker of gliomas, inhibits glioma cell proliferation and induces apoptosis. We further demonstrate that TRAF6 is a direct functional target of miR-146b-5p in gliomas and silencing of TRAF6 may mimic the above anti-tumor effects of miR-146b-5p. Our findings suggest miR-146b-5p and TRAF6 as potential therapeutic candidates for malignant gliomas.

## RESULTS

### miR-146b-5p is correlated with better prognosis in human gliomas

**Figure 1:**
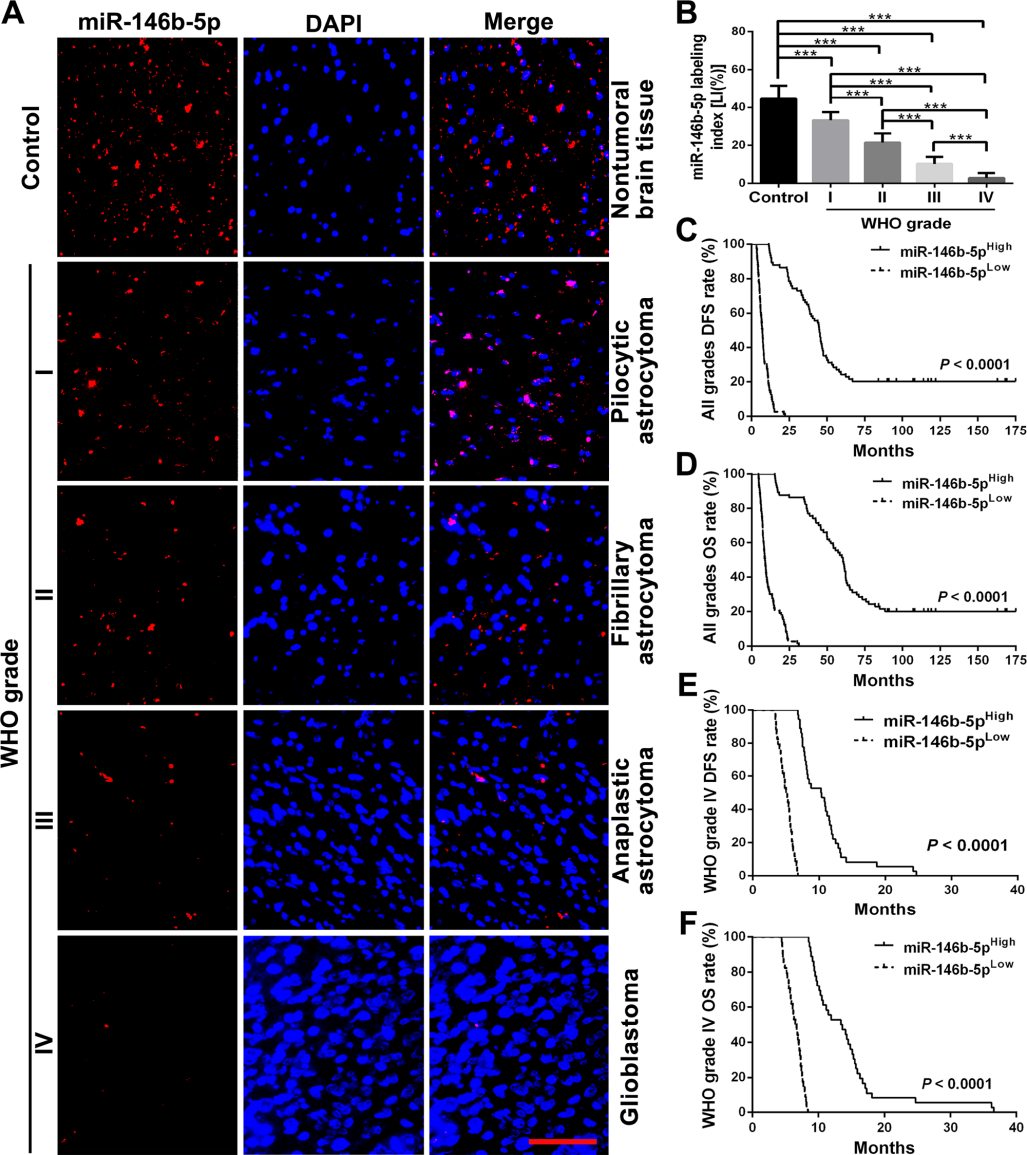
miR-146b-5p expression correlates with glioma grades and patients’ prognoses **A.** Representative images of miR-146b-5p ISH detection. Scale bar, 50 μm. **B.** Comparisons among groups of miR-146b-5p expression level [Labeling index, LI (%)] in the FFPE samples of 147 gliomas and 20 nontumoral control brain tissues. The miR-146b-5p LI (%) of each sample was calculated with Leica Image Pro Plus 5.0 software according to percentage ratio of positive cell number to total cell number and the data in B are presented as the mean ± SD. ***P < 0.001. **C–F.** Kaplan-Meier analysis of the correlation between miR-146b-5p and DFS or and OS of all grade glioma patients (C and D) and WHO grade IV (glioblastoma) patients (E and F). Patients were stratified into high and low expression subgroups using the median of miR-146b-5p LIs.

To identify relationships between miR-146b-5p expression in gliomas and histopathological grades or patients’ prognosis, ISH with LNA-modified probes was applied to detect endogenous miR-146b-5p expression in the FFPE specimens of 147 gliomas and 20 nontumoral brain tissues from human. We found that miR-146b-5p expression in gliomas was lower than that in the control (*P* < 0.001) and that its expression was significantly decreased with the elevation of glioma grades and was the lowest in glioblastoma (*P* < 0.001; Figure 1A and 1B). Kaplan-Meier analyses showed that the patients with higher level of miR-146b-5p had longer disease-free survival (DFS; *P* < 0.0001) and overall survival (OS; *P* < 0.0001; Figure 1C and 1D and Supplementary Figure S1A and S1B). Significantly, we found that glioblastoma patients could be divided into two subgroups with different outcomes based on miR-146b-5p expression, i.e., the higher expression of miR-146b-5p was, the better prognosis of patients (DFS: *P* < 0.0001; OS: *P* < 0.0001; Figure 1E and 1F). Both multivariate and univariate analyses showed that miR-146b-5p was an independent predictor for DFS and OS of glioma patients (Table 1 and Supplementary Table 1). These data indicate the inverse association of miR-146b-5p expression with glioma malignancy and reveal that miR-146b-5p is a potential prognostic biomarker for glioma patients.

**Table 1:** Multivariate analysis for DFS and OS in patients with gliomas

Factors	DFS		OS	
	HR (95%CI)	*P*	HR (95%CI)	*P*
Gender	1.033 (0.689–1.550)	0.8739	1.062 (0.705–1.599)	0.7741
Age	0.990 (0.975–1.006)	0.2114	0.991 (0.976–1.007)	0.2698
Predominant side	1.478 (1.309–2.102)	0.0298	1.475 (1.041–2.090)	0.0289
Predominant location	1.216 (1.035–1.429)	0.0177	1.255 (1.064–1.481)	0.0071
miR-146b-5p LI	0.678 (0.592–0.777)	<0.0001	0.676 (0.592–0.771)	<0.0001
TRAF6 LI	1.004 (0.911–1.106)	0.9412	0.976 (0.881–1.081)	0.5706
Ki-67 LI	1.870 (1.553–2.252)	<0.0001	1.980 (1.616–2.427)	<0.0001

Abbreviations: HR, hazard ratio; CI, confidence interval; LI, labeling index.

### TRAF6 is a direct target of miR-146b-5p in human glioma cells

**Figure 2:**
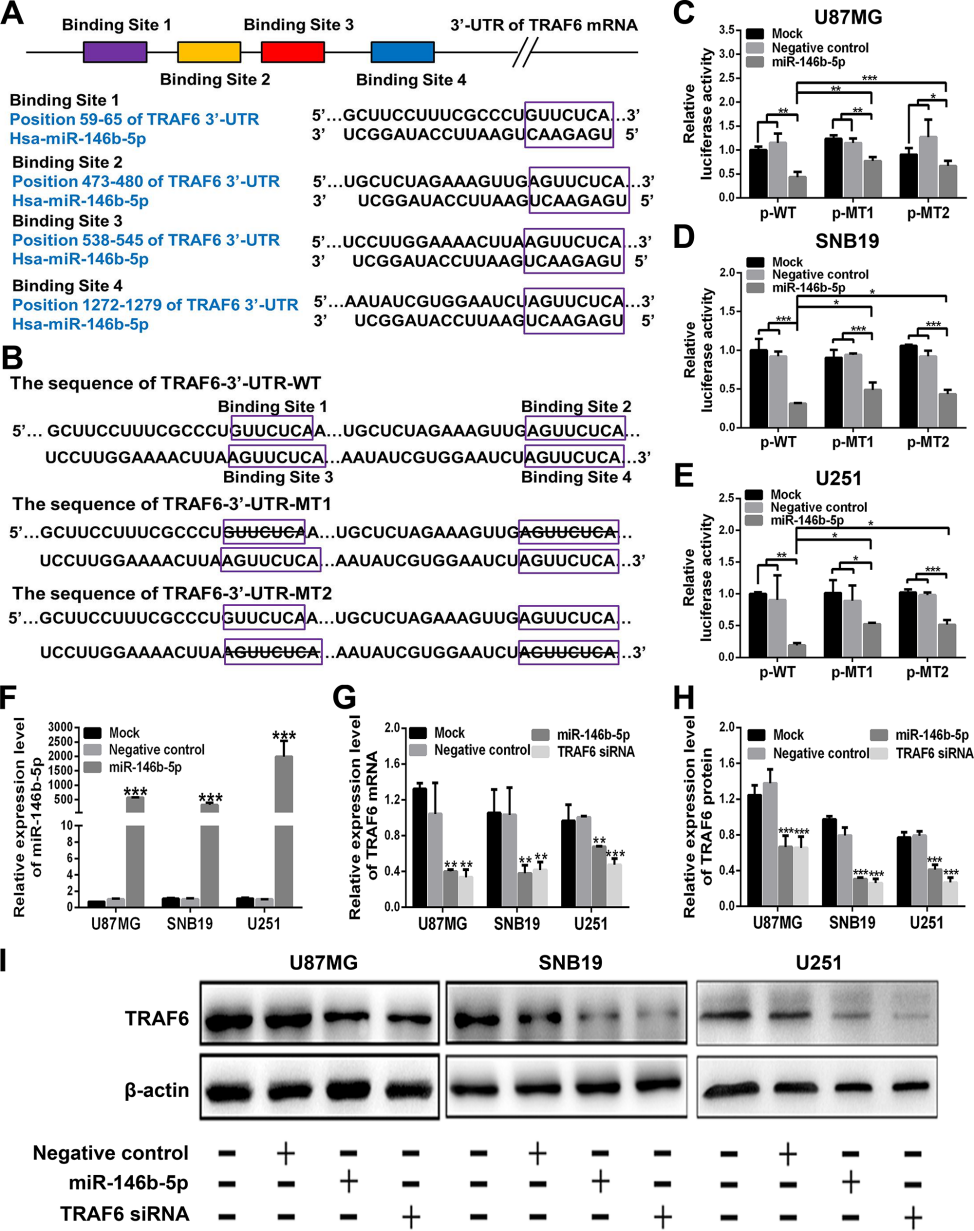
TRAF6 is a direct target of miR-146b-5p **A.** Four miR-146b-5p binding sites in TRAF6 3′-UTR predicted with TargetScan. **B.** Wild (TRAF6-3′-UTR-WT) and mutant (TRAF6-3′-UTR-MT1 and TRAF6-3′-UTR-MT2) TRAF6 3′-UTRs carried in recombinant luciferase mRNAs transcribed by p-WT, p-MT1 and p-MT2. The binding site 1 and 2 or 3 and 4 were deleted from TRAF6-3′-UTR-MT1 or TRAF6-3′-UTR-MT2. **C–E.** Luciferase reporter assays in U87MG (C), SNB19 (D) and U251 (E) cells transfected with p-WT, p-MT1 or p-MT2 (Mock), and cotransfected with p-WT, p-MT1 or p-MT2 and scrambled sequence (negative control) or miR-146b-5p mimics. **F** and **G.** qRT-PCR analyses of miR-146b-5p mimics-transfecting efficiency (F) and TRAF6 mRNA expression (G) in the cells as indicated. Their relative expression levels were normalized against U6 or GAPDH. The ratios of miR-146b-5p/U6 and TRAF6/GAPDH in untransfected cells (Mock) were arbitrarily set to 1.0. **H** and **I.** Western blot analysis of TRAF6 in the cells as indicated. The relative expression level of TRAF6 was normalized against β-actin. All experiments were performed at least in triplicate and the data in C - H are presented as the mean ± SD. *P < 0.05; **P < 0.01; ***P < 0.001.

TargetScan and miRTarBase predictions revealed that the 3′-UTR of TRAF6 mRNA contained four conserved miR-146b-5p binding sites (Figure 2A). To confirm the above predictions, we constructed three recombinant luciferase reporter vectors of TRAF6 3′-UTR, i.e., p-WT, p-MT1 and p-MT2. The recombinant luciferase mRNA transcribed by p-WT carried all miR-146b-5p binding sites (TRAF6-3′-UTR-WT) predicted in TRAF6 3′-UTR, while the one transcribed by p-MT1 or p-MT2 lacked the predicted binding site 1 and 2 (TRAF6-3′-UTR-MT1) or 3 and 4 (TRAF6-3′-UTR-MT2), respectively (Figure 2B). The dual-luciferase assay showed that miR-146b-5p could effectively suppress the luciferase activity delivered by the recombinant reporter vectors in glioblastoma cell lines (*P* < 0.05 ∼ 0.001) and that the effects in the cell lines cotransfected with miR-146b-5p and p-WT were stronger than those in the ones cotransfected with miR-146b-5p and p-MT1 or p-MT2 (*P* < 0.05 ∼ 0.001; Figure 2C–2E). To further verify whether miR-146b-5p directly knocked down TRAF6, we monitored the changes of miR-146b-5p and TRAF6 levels in the cell lines transfected with miR-146b-5p or TRAF6 siRNA by qRT-PCR and Western blotting. As shown in Figure 2F–2I, miR-146b-5p was significantly increased in miR-146b-5p-transfeced cell lines (*P* < 0.001; Figure 2F), but the mRNA and protein of TRAF6 were significantly decreased in the cell lines transfected with miR-146b-5p or TRAF6 siRNA (*P* < 0.01 ∼ 0.001; Figure 2G–2I), as compared with mock and negative control ones. The results reveal that miR-146b-5p may bind with TRAF6 3′-UTR and inhibit TRAF6 protein expression through inducing degradation of its mRNA in glioblastoma cells.

### TRAF6 overexpression is associated with miR-146b-5p downexpression and poorer prognosis in human gliomas

**Figure 3:**
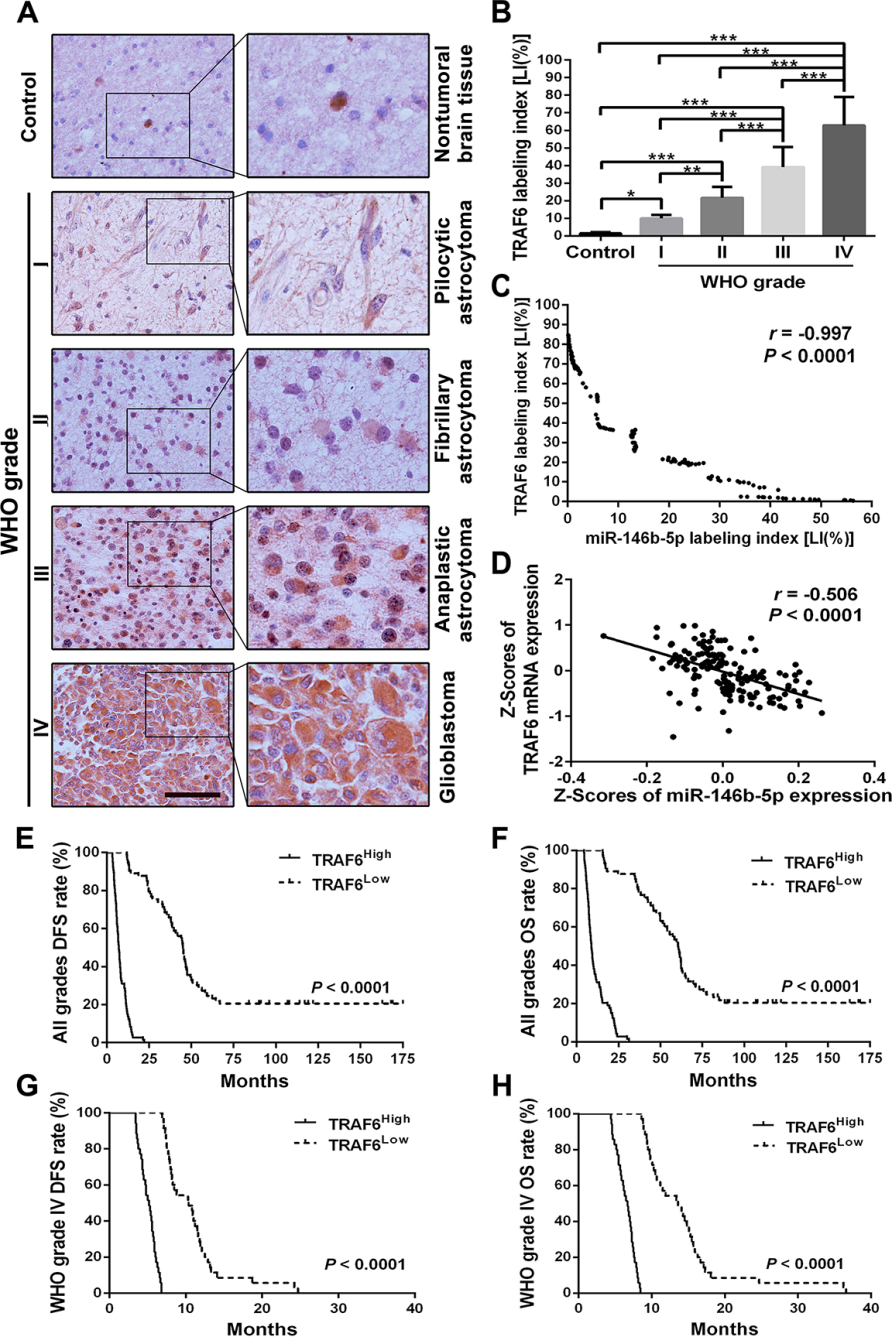
TRAF6 expression correlates with glioma grades, miR-146b-5p expression and patients’ prognoses **A.** Representative images of TRAF6 IHC detection. Scale bar, 50 μm. **B.** Comparisons among groups of TRAF6 expression level [Labeling index, LI (%)] in the FFPE samples of 147 gliomas and 20 nontumoral control brain tissues. The TRAF6 LI (%) of each sample was calculated according to percentage ratio of positive cell number to total cell number in 10 randomly selected microscopic fields at × 400, and the data in B are presented as the mean ± SD. *P < 0.05; **P < 0.01; ***P < 0.001. **C** and **D.** Pearson correlation analysis between TRAF6 and miR-146b-5p expressions in our FFPE samples (C) and the data from TCGA database (D) **E–H.** Kaplan-Meier analysis of the correlation between TRAF6 and DFS or and OS of all grade glioma patients (E and F) and glioblastoma patients (G and H). Patients were stratified into high and low expression subgroups using the median of TRAF6 LIs.

To investigate correlations between TRAF6 expression in gliomas and histopathological grades, miR-146b-5p expression or patients’ prognosis, IHC was used for detecting TRAF6 expression in the above FFPE specimens of gliomas and nontumoral brain tissues. We discovered that TRAF6 expression in gliomas was higher than that in the control (*P* < 0.05 ∼ 0.001) and that its expression was significantly increased with the elevation of glioma grades and was the highest in glioblastoma (*P* < 0.01 ∼ 0.001; Figure 3A and 3B). Moreover, TRAF6 expression in gliomas was negatively correlated with miR-146b-5p expression (*r* = −0.997, *P* < 0.0001; Figure 3C). The inverse relationship between the expressions of TRAF6 and miR-146b-5p was further validated by the glioblastoma data from TCGA database (*r* = −0.506, *P* < .0001; Figure 3D). Kaplan-Meier analyses demonstrated that the high level of TRAF6 expression predicted a short-term DFS (*P* < 0.0001) and OS (*P* < 0.0001; Figure 3E and 3F and Supplementary Figure S1C and S1D) in glioma patients. Moreover, glioblastoma patients could also be divided into two subgroups with different outcomes based on TRAF6 expression, i.e., the higher expression of TRAF6 was, the poorer prognosis of patients (DFS: *P* < 0.0001; OS: *P* < 0.0001; Figure 3G and 3H). Multivariate analysis showed that TRAF6 was not an independent predictor for DFS and OS of glioma patients (Table 1). However, univariate analysis demonstrated that TRAF6 could act as an auxiliary prognostic biomarker of patients’ survival (Supplementary Table 1). These data identify the positive correlation of TRAF6 expression with glioma malignancy, and further indicate that miR-146b-5p downexpression is an important cause resulting in TRAF6 overexpression in gliomas.

### miR-146b-5p inhibits cell proliferation and promotes apoptosis in human gliomas

**Figure 4:**
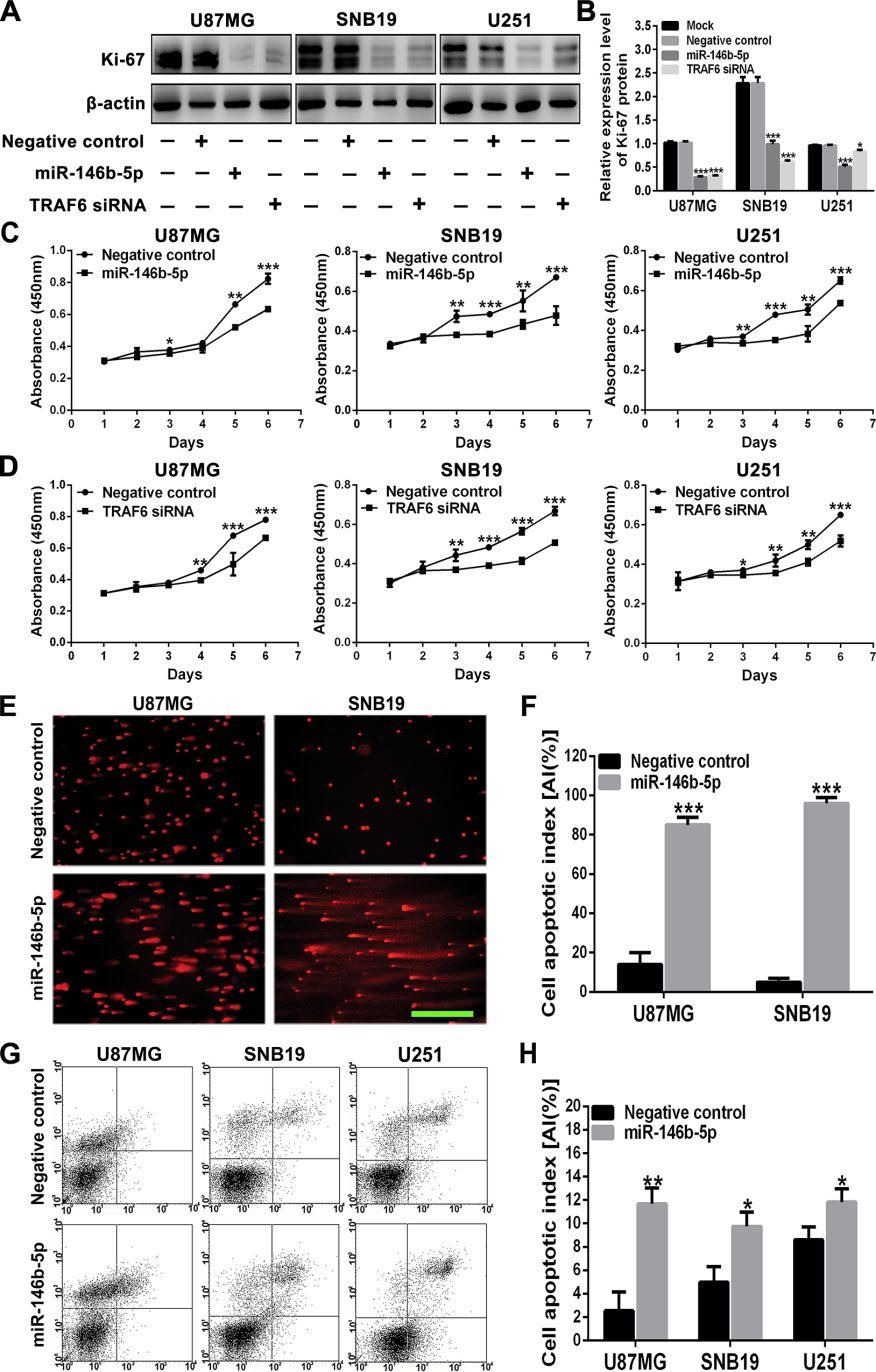
miR-146b-5p inhibits the proliferation of glioblastoma cells and promotes their apoptosis **A** and **B.** Western blot analysis (A) and comparisons among groups (B) of Ki-67 expression in U87MG, SNB19 and U251 cells untransfected (Mock) and transfected with scrambled sequence (negative control), miR-146b-5p mimics or TRAF6 siRNA. The relative expression level of Ki-67 was normalized against β-actin. **C** and **D.** Growth curves from the above transfected cells assessed by CCK8 assay. **E** and **F.** Representative images of comet cells (E) and apoptotic indexes (AIs; F) in the cells as indicated assessed by SCGE. Scale bar, 100 μm. **G** and **H.** Representative images (G) and AIs (H) in the cells as indicated assessed by FCM. AIs were calculated as mentioned in Materials and Methods. All experiments were performed at least in triplicate and the data in B - D, F and H are presented as the mean ± SD. *P < 0.05; **P < 0.01; ***P < 0.001.

To ascertain the associations of cell proliferation with tumor grades or the expressions of miR-146b-5p and TRAF6 in gliomas, we detected the expression of proliferation marker Ki-67 in the above FFPE specimens by IHC. In comparison with control brain tissues and grade I and II gliomas, Ki-67 LI was significantly increased in grade III gliomas (*P* < 0.05 ∼ 0.001) and was the highest in glioblastoma (*P* < 0.001; Supplementary Figure S2A and S2B). Pearson analysis confirmed that Ki-67 LIs were negatively correlated with miR-146b-5p LIs (*r* = −0.980, *P* < 0.0001; Supplementary Figure S2C) and positively correlated with TRAF6 LIs (*r* = 0.984, *P* < 0.0001; Supplementary Figure S2D) in gliomas. Through *in vitro* studies, we found that both transfected miR-146b-5p and TRAF6 siRNA significantly reduced the Ki-67 expression (*P* < 0.05∼0.001) and proliferation (*P* < 0.05∼0.001) of glioblastoma cell lines, as measured by Western blotting (Figure 4A and 4B) and CCK8 proliferation assay (Figure 4C and 4D). Moreover, transfection of miR-146b-5p also dramatically increased the apoptosis of glioblastoma cell lines (*P* < 0.05∼0.001), as measured by SCGE (Figure 4E and 4F) and FCM assay (Figure 4G and 4H). Our results reveal that miR-146b-5p downexpression and TRAF6 overexpression are responsible for excessive proliferation and reduced apoptosis of glioma cells, whereas both upregulation of miR-146b-5p and knockdown of TRAF6 may effectively reverse the malignant phenotype of glioblastoma cells.

### miR-146b-5p inhibits glioma malignant progression by blocking TRAF6-TAK1 pathway

**Figure 5:**
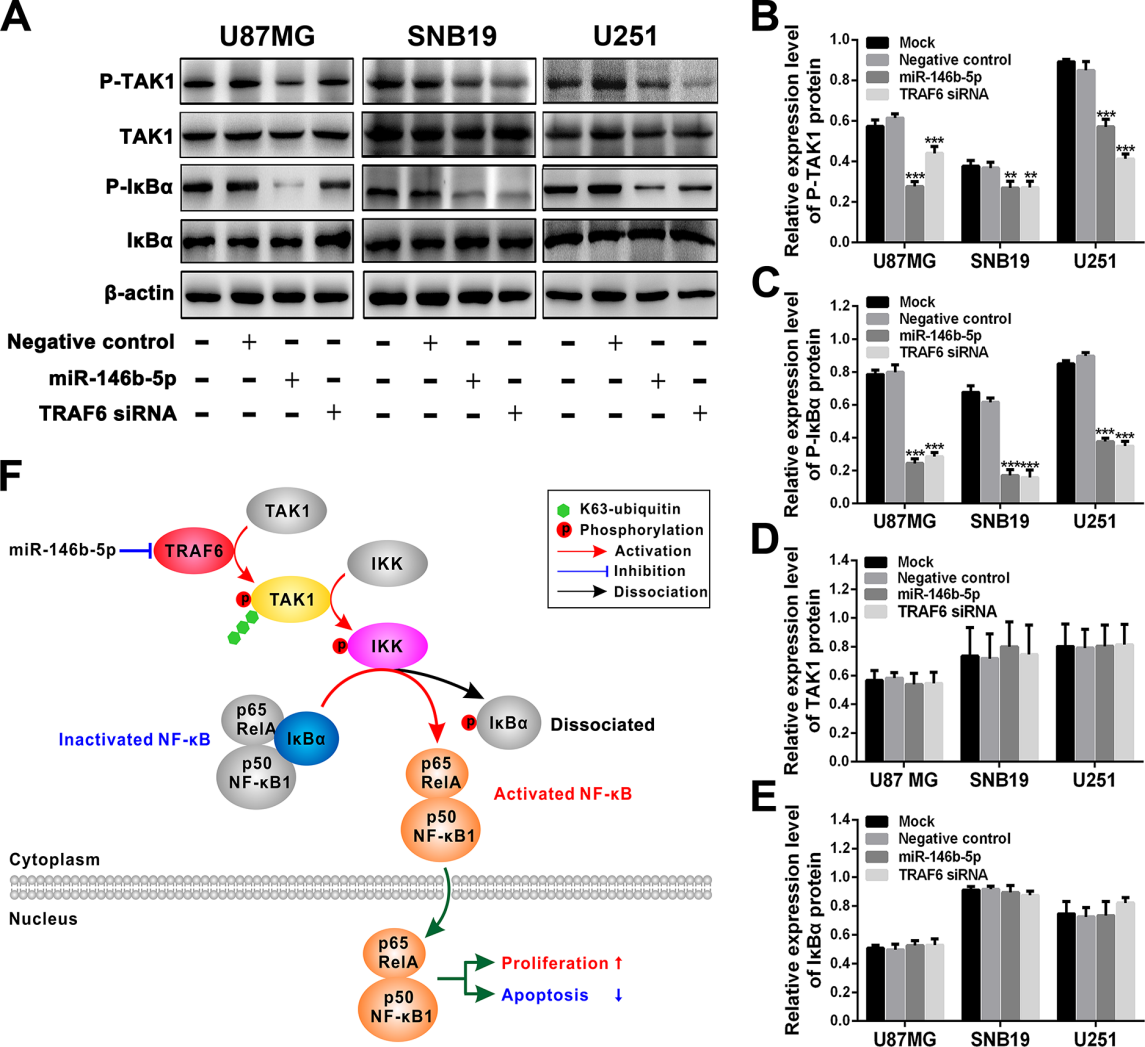
miR-146b-5p and TRAF6 siRNA suppress the activation of TRAF6-TAK1 pathway **A.** Western blot analyses of P-TAK1, TAK1, P-IκBα and IκBα in U87MG, SNB19 and U251 cells untransfected (Mock) and transfected with scrambled sequence (negative control), miR-146b-5p mimics or TRAF6 siRNA. **B–E**. Comparisons among groups of P-TAK1 (B), P-IκBα (C), TAK1 (D) and IκBα (E) expressions in the cells as indicated. The relative expression levels of the indicated proteins were normalized against β-actin. All experiments were performed at least in triplicate and the data in B - E are presented as the mean ± SD. **P < 0.01; ***P < 0.001. **F.** Schematic illustration of the molecular pathway by which miR-146b-5p inhibits the proliferation of glioblastoma cells and promotes their apoptosis.

To further determine the underlying mechanisms by which miR-146b-5p suppresses gliomas, we focused on TAK1 and IκBα, the important downstream effectors of TRAF6, to investigate whether they were responsible for the suppressed proliferation and increased apoptosis induced by miR-146b-5p. As shown in Figure 5, phosphorylated TAK1 and IκBα (P-TAK1 and P-IκBα) were significantly reduced in three glioblastoma cell lines transfected with miR-146b-5p or TRAF6 siRNA as compared with mock and negative control ones (*P* < 0.01 ∼ 0.001; Figure 5A–5C), but the expressions of TAK1 and IκBα remained constant among four groups of the three cell lines (*P* > 0.05; Figure 5A, and 5E). The results reveal that miR-146b-5p suppresses the activation of TRAF6-TAK1 pathway by directly targeting TRAF6, and then decreased IκBα phosphorylation and NF-κB activation, thereby inhibiting glioma cells proliferation and inducing apoptosis (Figure 5F). It further verifies the dependability of the above results that TRAF6 siRNA imitates the effects of miR-146b-5p.

## DISCUSSION

Previous reports have showed that miR-146b-5p may function as a tumor suppressor or promoter in different tumors [18–24]. However, the clinical relevance of miR-146b-5p in gliomas remains unknown. In the present study, we identified miR-146b-5p as a tumor suppressor to inhibit proliferation and promote the apoptosis in astrocytic gliomas, representing the first comprehensive analysis of miR-146b-5p in gliomas. Mechanistically, we verified TRAF6 as a direct functional target of miR-146b-5p, which facilitated our understanding of the mechanisms underlying glioma malignant progression. Most importantly, we found that miR-146b-5p and TRAF6, not only were correlated with each other, but also predicted the survival of glioma patients, highlighting the potential values of miR-146b-5p and TRAF6 as novel prognostic biomarkers in human gliomas.

The integrated analyses based on miRNAs and their regulatory networks have provided new ideas for searching clinical biomarkers correlating with glioma grades, specific subtypes or prognosis, highlighting the potential values of miRNAs and their target proteins in diagnosis and subclassification [28–30]. Our present data demonstrated that miR-146b-5p expression was significantly decreased and TRAF6 expression was significantly increased with the grade elevation in 15 grade I glioma, 28 grade II glioma, 34 grade III glioma, and 70 glioblastoma (grade IV) specimens, suggesting that miR-146b-5p and TRAF6 were the potential biomarkers in distinguishing glioma grades. The glioma subgroups with higher level of miR-146b-5p and lower level of TRAF6 were correlated with better prognoses, suggesting that miR-146b-5p and TRAF6 were the specific biomarkers for the prognostic-based glioma subclassification. There was an inverse correlation between the expressions of miR-146b-5p and TRAF6, implying that miR-146b-5p downexpression was an important cause leading to TRAF6 overexpression in gliomas. Furthermore, miRNAs stably exist in FFPE samples and can be easily detected by ISH [27, 31]. Thus, miR-146b-5p and TRAF6 could be the novel and clinical feasible candidates for diagnosis and subclassification for gliomas.

Malignant gliomas, of which 60 to 70% are glioblastoma, are characterized by rapid growth and relentless invasion [3, 32]. However, the unlimited proliferation and reduced apoptosis of tumor cells are the important causes resulting in the rapid growth and invasion of malignant gliomas [3, 32]. In our glioma tissue specimens, the Ki-67 LIs (proliferation indexes) of malignant gliomas (grade III and IV) were significantly higher than those of grade I and II gliomas (Supplementary Figure S2A and S2B), and Ki-67 LIs were negatively correlated with miR-146b-5p LIs and positively correlated with TRAF6 LIs (Supplementary Figure S2C and S2D), suggesting that miR-146b-5p downexpression and TRAF6 overexpression were the important causes leading to the excessive proliferation of malignant glioma cells. Our *in vitro* results showed that both miR-146b-5p and TRAF6 siRNA could effectively decrease the Ki-67 expression and proliferation of glioblastoma cells, and miR-146b-5p also significantly promoted the apoptosis of glioblastoma cells. These facts further confirmed that miR-146b-5p as a tumor suppressor inhibited cell proliferation and promoted apoptosis in malignant gliomas, and suggested that miR-146b-5p exerted the above effects at least partly by blocking TRAF6 expression, highlighting the potential values of miR-146b-5p and TRAF6 in the therapy of malignant gliomas.

TRAF6, which functions as an E3 ubiquitin ligase, has recently been discovered to promote oncogenesis through activating NF-κB [33–35]. The dimer p65RelA: p50NF-κB1 (p65:p50) is the main NF-κB subtype which accelerates cell proliferation and restrains apoptosis [35–38]. IκBα binds with p65:p50 and inhibits p65:p50 activation and nuclear translocation [36]. TRAF6 catalyzes the synthesis of K63-linked polyubiquitin chain which recruits TAK1 and IKK, and induces the autophosphorylated activation of TAK1 and the phosphorylated activation of IKK catalyzed by P-TAK1, while P-IKK makes IκBα dissociate from p65:p50 by catalyzing IκBα phosphorylation, thereby resulting in the activation and nuclear translocation of p65:p50 [39–41]. We identified TRAF6 as a direct functional target of miR-146b-5p by bioinformatics prediction and luciferase reporter assay. Subsequently, we confirmed that miR-146b-5p-induced suppression of TRAF6 could inactivate TAK1 pathway and reduce IκBα phosphorylation, and that siRNA knockdown of TRAF6 could perfectly imitate the above effects of miR-146b-5p. Combining with the inverse relevance of miR-146b-5p and TRAF6 expressions in the glioma specimens, our results indicated that TRAF6 overexpression induced by miR-146b-5p downexpression promoted the cell proliferation and inhibited apoptosis in malignant gliomas by activating TAK1 pathway and then accelerating the phosphorylated dissociation of IκBα from p65:p50, which enhanced our comprehension of the molecular mechanism of glioma malignant progression (Figure 5F) and suggested that miR-146b-5p could be potentially applied in the treatment of malignant gliomas.

Our study showed that miR-146b-5p expression was decreased in gliomas, especially in glioblastoma. The miR-146b-5p precursor gene (hsa-miR-146b-5p) is located on chromosome 10q24-26 region frequently lost in gliomas [27, 42–44]. Our previous study has identified the reduction of miR-146b-5p expression caused by 10q loss in human glioblastoma specimens [27]. However, the molecular mechanism of miR-146b-5p downregulation remains unknown in gliomas without 10q loss. Further studies are underway to investigate the unknown mechanism. Our multivariate analysis showed that TRAF6 was not an independent prognostic factor for DFS and OS of glioma patients, but univariate analysis demonstrated that TRAF6 could act as an auxiliary prognostic biomarker of patients’ survival. Since other target genes, such as MMP16 and EGFR [27, 45, 46], were also involved in the modulation induced by miR-146b-5p, the prognostic significance of TRAF6 was not as important as that of miR-146b-5p in gliomas.

In summary, our study revealed that miR-146b-5p inhibited the proliferation of glioma cells and promoted their apoptosis *in vitro* and *in vivo* by directly targeting TRAF6, and predicted better prognosis in human gliomas, especially in glioblastoma. Its downregulation was an important cause resulting in gliomagenesis and malignant progression. More importantly, miR-146b-5p might be a novel biomarker for molecular subclassification of malignant gliomas and a therapeutic candidate for these lethal diseases.

## MATERIALS AND METHODS

### Tissue samples and clinical data

The surgical specimens of 147 astrocytic gliomas and 20 nontumoral brain tissues (control) were collected in Tianjin Medical University General Hospital (TMUGH) with written consent. After surgical excision, specimens were immediately fixed in 3.7% buffered formaldehyde solution. The formalin-fixed, paraffin-embedded (FFPE) samples were stored at room temperature. The FFPE tissue sections of 5 μm thickness were cut for HE staining, miR-146b-5p *in situ* hybridization and the immunohistochemical detections of TRAF6 and Ki-67. Pathological diagnoses were independently made by two neuropathologists according to the 2007 World Health Organization (WHO) classification of central nervous system tumors [47]. The WHO grades and patients’ clinical features of these gliomas were summarized in Supplementary Table 2. All of the 147 glioma patients had complete information and were followed up after operation until December 31, 2013, with a follow-up time of 4.5 months to 15 years. This study was carried out in accordance with the principles of the Helsinki Declaration and approved by the Ethics Committee of TMUGH.

An independent cohort of 158 patient specimens from The Cancer Genome Atlas (TCGA) database (https://tcgadata.nci.nih.gov/tcga) was used to validate the correlation between the expressions of miR-146b-5p and TRAF6 in glioblastoma.

### Locked nucleic acid-modified oligonucleotide probes

Locked nucleic acid (LNA)-modified oligonucleotide probes labeled with digoxin at their 5′ends were synthesized by TaKaRa. The sequences of miR-146b-5p probe and scramble control probe were 5′-ALgCCTLaTGGLaATTLcAGTLtCTCA-3′ and 5′-CLgTAT LaGGCLcCAALgAATLtAGG-3′, respectively. La, Lt, Lc, and Lg were LNA monomers corresponding to the bases A, T, C, and G.

### *In situ* hybridization (ISH) with LNA-modified oligonucleotide probes

FFPE tissue sections adhered to glass slides were deparaffinized in xylene and rehydrated through gradient ethanol. The slides were then digested with 0.4% pepsin (Sigma) at 37°C for 20 min, washed thrice in PBS containing 0.2% glycine and blocked with 100 μl prehybridization solution (Boster) at 37°C for 3 hours. The slides were then hybridized in a ThermoBrite (Abbott Molecular) overnight at 37°C, using 5.0 μg/ml LNA-modified probes diluted with miRNA ISH solution (Boster). After hybridization, slides were washed four times in 50% deionized formamide/2 × SSC at 37°C, washed for 5 min at 37°C in PBS. The slides were incubated with anti-digoxin Rhodamine (TRITC) -conjugated antibody (Roche) in humidified chambers overnight at 4°C, followed by four washes in PBS. The cell nuclei were counterstained with 4′, 6-diamidino-2-phenylindole (DAPI; Roche). The hybridization images were acquired under a DM600B fluorescent microscope (Leica) and the percentage ratio [Labeling index (%), LI] of positive cell number to total cell number was calculated with Image Pro Plus 5.0 software (Leica).

### Cell lines and cell culture

Human glioblastoma cell lines, U87MG, SNB19 and U251 cells, were used for present study. U87MG was obtained from the American Type Culture Collection, SNB19 and U251 were purchased from the China Academia Sinica Cell Repository (Shanghai). U87MG, SNB19 and U251 cells were cultured in Dulbecco's Modified Eagle Medium (DMEM; Gibco) containing 10% fetal bovine serum (Gibco). All cell lines were incubated at 37°C in a humidified incubator with 5% CO_2_ / 95% air.

### Luciferase plasmid construction

The candidate target genes of miR-146b-5p were predicted using miRTarBase (http://mirtarbase.mbc.nctu.edu.tw/) and TargetScan (http://www.Targetscan.org/). The pEZX-MT01 Luciferase miRNA Expression Reporter Vector (GeneCopoeia) was applied to construct the wild (p-WT) and mutant (p-MT1 and p-MT2) reporter vectors of TRAF6 3′-untranslated region (3′-UTR). The primer pair used for TRAF6 3′-UTR cDNA amplification has been listed in Supplementary Table 3. The coding-sequences of predicted mR-146b-5p binding site 1 and 2 or 3 and 4 were respectively deleted from TRAF6 3′-UTR cDNA used to construct p-MT1 or p-MT2 by site-directed mutagenesis. The sequences and orientations of the inserts in three vectors were validated by DNA sequencing.

### Dual-luciferase reporter assay

U87MG, SNB19 and U251 cells seeded in 96-well plates were cotransfected with 0.15 μg p-WT, p-MT1 or p-MT2 and 0.08 μg miR-146b-5p mimics or scrambled sequence (negative control) using X-tremeGENE siRNA Transfection Reagent (Roche). The mock controls were set up by transfecting the three cell lines only using the above recombinant reporter vectors. The activities of renilla and firefly luciferases were detected with Dual-Luciferase Reporter Assay System (Promega) on a Synergy 2 Microplate Reader Fluorometer (BioTek). The results were presented after normalization with the measured values of firefly luciferase.

### miR-146b-5p mimics and TRAF6 siRNA transfection

The dsRNA oligonucleotides of miR-146b-5p mimics, small interfering RNA silencing TRAF6 (TRAF6 siRNA) and scrambled sequence (negative control) were purchased from GenePharma. Their sequences have been listed in Supplementary Table 4. The U87MG, SNB19 and U251 cells of miR-146b-5p group, TRAF6 siRNA group and negative control group were respectively transfected with the corresponding dsRNA oligonucleotides using X-tremeGENE siRNA Transfection Reagent (Roche). The mock groups of the above cell lines were treated only using the transfection reagent of the same volume.

### Quantitative RT-PCR (qRT-PCR)

Total RNA from each group of the three cell lines was extracted using TRIzol reagent (Invitrogen). miR-146b-5p was quantified with Stem-loop qRT-PCR Detection Kit (GenePharma). Reverse Transcription System and GoTaq qPCR Master Mix Kit (Promega) were used for the qRT-PCR detection of TRAF6 mRNA. U6 and glyceraldehyde-3-phosphate dehydrogenase (GAPDH) were used as the internal controls of miR-146b-5p and TRAF6 mRNA, respectively. Specific primers for TRAF6 mRNA detection have been listed in Supplementary Table 5. All reactions were performed on a CFX Connect™ Real-Time PCR Detection System (Bio-Rad). The fold changes of miR-146b-5p and TRAF6 mRNA levels were calculated by the 2^−ΔΔCt^ method.

### Western blotting

Western blotting was carried out as previously described [27]. The primary antibodies used in this study were as follows: rabbit anti-Ki-67, rabbit anti-TRAF6 (Millipore), rabbit anti-TAK1, rabbit anti-phospho-TAK1, rabbit anti-IκBα, mouse anti-phospho-IκBα (Cell Signaling Technology), and mouse anti-β-actin (Boster).

### Immunohistochemistry (IHC)

The IHC staining of TRAF6 and Ki-67 was performed using VECTASTAIN ABC Detection System (including biotinylated goat anti-rabbit IgG secondary antibody; VECTOR). The whole process was conducted as the manufacturer's instructions. Primary antibodies included rabbit anti-TRAF6 and rabbit anti-Ki-67 (Millipore). The results of TRAF6 and Ki-67 detections were independently evaluated by two neuropathologists. Their LIs were determined according to the percentage of positive cell number to total cell number in 10 randomly selected microscopic fields at × 400.

### Tumor cell proliferation assay (CCK8 assay)

U87MG, SNB19 and U251 cells (800 cells per well) were seeded into 96-well plates. At 1, 2, 3, 4, 5 and 6 days after transfecting miR-146b-5p mimics, TRAF6 siRNA or scrambled sequence (negative control), 20 μL of Cell Counting Kit-8 (Beyotime) were added to each well and incubated for 2 hours at 37°C. The absorbance at 450 nm was measured on a Synergy 2 microplate reader (BioTek).

### Alkaline single cell gel electrophoresis (SCGE)

The U87MG and SNB19 cells of miR-146b-5p and negative control groups were harvested at 48 hours after transfection. The cells (1 × 10^4^) of two groups per cell line were mixed with 0.6% (w/v) low melting agarose in PBS (pH 7.4) at 37°C and immediately layered on the frosted glass slides previously coated with a layer of 0.75% (w/v) normal melting agarose in PBS (pH 7.4). SCGE was carried out as previously described [48]. After electrophoresis, the slides were stained with ethidium bromide (50 μg / ml) and the SCGE images were acquired under a DM600B fluorescent microscope (Leica). The Comet Assay IV software program (Perceptive) was used to identify apoptotic cells and calculate the percentage ratio [Apoptotic index (%), AI] of apoptotic cell number to total cell number.

### Flow cytometry assay (FCM)

U87MG, SNB19 and U251 cells of miR-146b-5p and negative control groups were harvested at 48 hours after transfection and stained with fluorescein isothiocyanate- conjugated annexin V (annexin V-FITC) and propidium iodide (PI). The staining procedure was conducted with Annexin V-FITC Apoptosis Detection Kit (KeyGEN) as the manufacturer's instructions. Bioscience FACScan Flow Cytometry System (BD) was used to detect apoptotic cells and calculate the AIs of two groups per cell line.

### Statistical analyses

All statistical analyses were performed with SPSS 18.0 software. Data were presented as the mean ± standard deviation (SD). The normality of distributions was estimated using Kolmogorov-Smirnov test. The differences among/between sample groups were analyzed by one-way ANOVA or Student *t* test. The Pearson correlation analysis was used to determine the correlations between miR-146b-5p and TRAF6 (including the data from TCGA database), miR-146b-5p and Ki-67 or TRAF6 and Ki-67. Patients’ survival was analyzed with Kaplan-Meier method and compared with log-rank test. The median was used to determine the cutoff in the glioma cohort from all grades, and WHO grade I∼II, III and IV groups. The Cox's proportional hazards regression model was applied for univariate and multivariate survival analyses. Statistical significance was assigned at *P* < 0.05 (*), *P* < 0.01 (**) or *P* < 0.001 (***). All the experiments of cell lines were performed at least three times with triplicate samples.

## SUPPLEMENTARY FIGURES AND TABLES


