# Supplementary material for: miR-146b-5p functions as a tumor suppressor by targeting TRAF6 and predicts the prognosis of human gliomas
Source: Oncotarget. 2015 Aug 6;6(30):29129–42. doi: 10.18632/oncotarget.4895 (PMC4745716; doi:10.18632/oncotarget.4895)
Supplement: Supplementary file 1 [file oncotarget-06-29129-s001.pdf]

SUPPLEMENTARY FIGURES AND TABLES

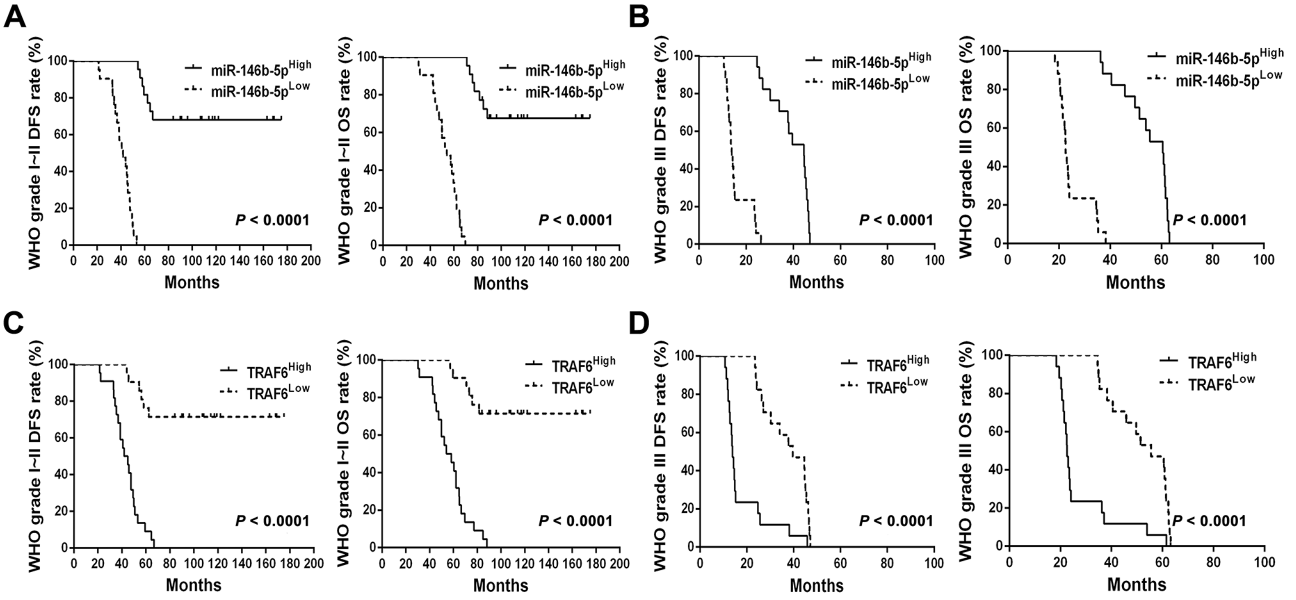

Supplementary Figure S1: miR-146b-5p and TRAF6 expressions correlate with patients' prognoses. A and **B.** Kaplan-Meier analysis of the correlation between miR-146b-5p and DFS (left) or OS (right) of WHO grade I~II glioma patients (A) and WHO grade III glioma patients (B) **C** and **D.** Kaplan-Meier analysis of the correlation between TRAF6 and DFS (left) or OS (right) of WHO grade I~II glioma patients (C) and WHO grade III glioma patients (D) The patients of different grade groups were stratified into high and low expression subgroups using the median of miR-146b-5p LIs or TRAF6 LIs.

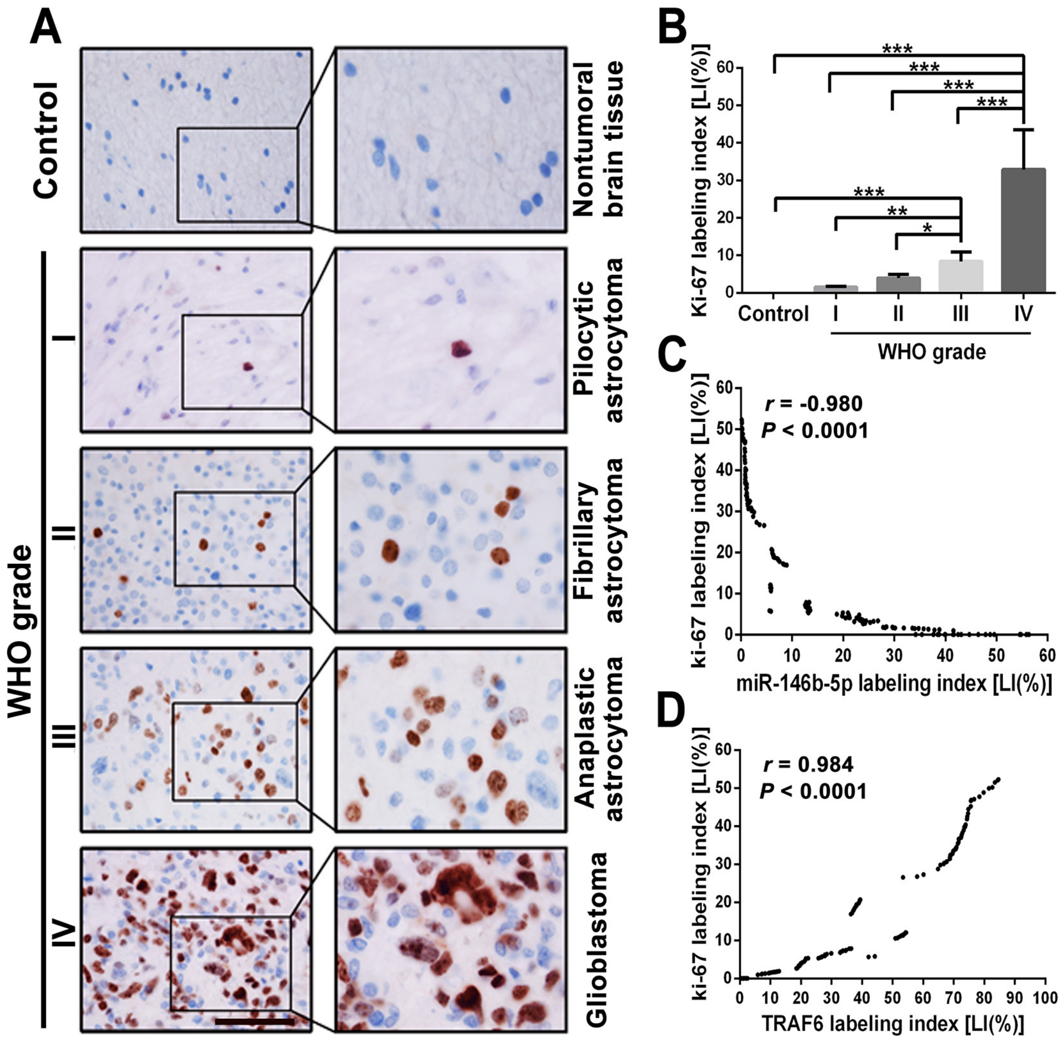

Supplementary Figure S2: Ki-67 expression correlates with glioma grades and their expressions of miR-146b-5p and TRAF6. A. Representative images of Ki-67 IHC detection. Scale bar, 50 μm. **B.** Comparisons among groups of Ki-67 labeling index [LI (%)] in the FFPE samples of 147 gliomas and 20 nontumoral control brain tissues. The Ki-67 LI (%) of each sample was calculated according to percentage ratio of positive cell number to total cell number in 10 randomly selected microscopic fields at × 400, and the data in B are presented as the mean ± SD. *$P < 0.05$; **$P < 0.01$; ***$P < 0.001$. **C** and **D.** Pearson correlation analysis between Ki-67 LIs and miR-146b-5p LIs (C) or TRAF6 LIs (D) in the FFPE samples as indicated.

Supplementary Table S1: Univariate analysis for DFS and OS in patients with gliomas.

Factors	DFS		OS	
	HR (95%CI)	*P*	HR (95%CI)	*P*
Gender	1.307 (0.921–1.855)	0.1314	1.319 (0.929–1.872)	0.1218
Age	1.050 (1.038–1.061)	<0.0001	1.049 (1.038–1.061)	<0.0001
Predominant side	0.787 (0.604–1.027)	0.0775	0.787 (0.603–1.026)	0.0764
Predominant location	1.507 (1.341–1.693)	<0.0001	1.509 (1.343–1.696)	<0.0001
miR-146b-5p LI	0.596 (0.536–0.663)	<0.0001	0.638 (0.582–0.699)	<0.0001
TRAF6 LI	1.224 (1.185–1.264)	<0.0001	1.214 (1.176–1.254)	<0.0001
Ki-67 LI	1.489 (1.402–1.582)	<0.0001	1.518 (1.427–1.615)	<0.0001

Abbreviations: HR, hazard ratio; CI, confidence interval; LI, labeling index.

Supplementary Table S2: The clinical features of the glioma specimens used in this study.

Feature	WHO Grade			
	I (*n* = 15)	II (*n* = 28)	III (*n* = 34)	IV (*n* = 70)
Gender				
Male	7	16	18	46
Female	8	12	16	24
Age (Year, Mean ± SD)	13 ± 6.9	41 ± 12.2	46 ± 14.7	60 ± 12.1
Predominant side				
Left	6	13	15	38
Right	5	13	17	31
Middle	4	2	2	1
Predominant location				
Frontal lobe	0	23	23	32
Temporal lobe	0	2	5	26
Parietal lobe	0	1	4	5
Occipital lobe	0	0	1	5
Pineal body	1	0	0	0
Saddle area	1	0	0	0
Thalamus	1	0	0	0
Cerebellum	12	2	0	2
Third ventricle	0	0	1	0

Abbreviation: SD, Standard deviation.

Supplementary Table S3: Primers used for TRAF6 3′-UTR RT-PCR amplification.

Primers	Sequence
Forward	5′-GCGATCGCTATATGTAATATATTAAAAGTGAAA-3′
Reverse	5′-GGAGCTCAAATAATTAAGGTTATATTTAGG-3′

Supplementary Table S4: miR-146b-5p, TRAF6 siRNA and Negative control.

dsRNA	Sequence
miR-146b-5p mimics	5′-UGAGAACUGAAUUCCAUAGGCU-3′
	3′-UUACUCUUGACUUAAGGUAUCC-5′
TRAF6 siRNA	5′-GCCUAAUCAUUAUGAUCUATT-3′
	3′-TTCGGAUUAGUAAUACUAGAU-5′
Negative control	5′-UUCUCCGAACGUGUCACGUTT-3′
	3′-TTAAGAGGCUUGCACAGUGCA-5′

Supplementary Table S5: Primers used for TRAF6 mRNA qRT-PCR detection.

Primers	Sequence	
TRAF6	forward	5′-TGCTTGATGGCATTACGAGAA-3′
	reverse	5′-CATTTGGACATTTCACCATCAGAG-3′
GAPDH	forward	5′-TGCACCACCAACTGCTTAGC-3′
	reverse	5′-GGCATGGACTGTGGTCATGAG-3′
